# Acupuncture combined with rehabilitation robot for recovery of hemiplegia after ischemic stroke: systematic review and meta-analysis of randomized controlled trials

**DOI:** 10.3389/fneur.2026.1789103

**Published:** 2026-04-10

**Authors:** Geng Li, Quan Wang, Li Li, Yu Xu, Xin Zhao, Shuangli Liu, Lin Cong

**Affiliations:** 1 The First Clinical Medical College, Yunnan University of Chinese Medicine, Kunming, China; 2 Yunnan Provincial Hospital of Traditional Chinese Medicine, Kunming, China; 3 Yunnan University of Chinese Medicine, Kunming, China

**Keywords:** acupuncture therapy, brain ischemia, hemiplegia, recovery of function, robotics, stroke rehabilitation

## Abstract

**Introduction:**

Effective rehabilitation for persistent post-stroke motor impairment is a major clinical need. The efficacy of combining acupuncture with robot-assisted therapy for hemiplegia is unclear. This study aimed to evaluate the efficacy of combining acupuncture with rehabilitation robot (RR) for improving limb function in patients with hemiplegia after ischemic stroke.

**Methods:**

This systematic review was conducted according to PRISMA guidelines. Eight electronic databases were searched up to November 29, 2025, for randomized controlled trials (RCTs) comparing combined acupuncture and RR therapy with control interventions in adults in the subacute phase of hemiplegia after ischemic stroke. Primary outcomes were motor function (Fugl-Meyer Assessment) and activities of daily living (Barthel Index). The risk of bias was assessed with the Cochrane RoB 2.0 tool. Meta-analyses were performed using R software (version 4.5.1).

**Results:**

Twenty RCTs with 1,594 patients were included. Across all outcomes, combination therapy consistently proved superior: Fugl-Meyer Assessment of Upper Extremity [FMA-UE, mean difference (MD) = 9.80, 95% Confidence Interval (CI): 8.04–11.56], Fugl-Meyer Assessment of Lower Extremity (FMA-LE, MD = 4.00, 95% CI: 2.79–5.21), Barthel Index (BI, MD = 8.29, 95% CI: 6.62–9.95), and Effective Rate (risk ratio = 1.17, 95% CI: 1.08–1.27). Sensitivity analyses confirmed robust findings for FMA-UE, FMA-LE, and BI. Subgroup analysis revealed that baseline motor function contributed to heterogeneity; however, patients with poorer baseline function (FMA-UE < 20) consistently experienced a greater benefit. The intervention demonstrated a favorable safety profile.

**Conclusion:**

Acupuncture combined with RR is an effective and safe intervention for improving motor function and daily living activities in patients with post-ischemic stroke hemiplegia.

**Systematic review registration:**

https://www.crd.york.ac.uk/PROSPERO/view/CRD420251155831, Identifier: CRD420251155831.

## Highlights

- Acupuncture combined with rehabilitation robot therapy significantly improves limb function after ischemic stroke.
- Acupuncture combined with rehabilitation robot therapy enhances daily living activities in individuals with post-stroke hemiplegia.
- Patients with severe baseline upper limb impairment benefit most consistently.
- A robust meta-analysis of 20 RCTs confirms efficacy and a favorable safety profile.

## 1 Introduction

Ischemic stroke (IS) remains a leading cause of disability worldwide (1). Although acute-phase reperfusion therapies, such as thrombolysis and endovascular thrombectomy, improve outcomes (2), functional impairment remains a major burden. For example, among patients receiving endovascular therapy, only 20–30% achieve full or favourable motor recovery, while up to 50–60% continue to have limb dysfunction 6 months after onset (3, 4). These deficits severely limit daily activities and quality of life, while also imposing significant burdens on families and the broader socioeconomic context (5). Consequently, finding better rehabilitation strategies for post-stroke limb dysfunction is a key clinical priority and challenge.

Conventional rehabilitation methods—such as balance pad training, weight-shifting exercises, and task-oriented gait training—can improve motor function after stroke, yet they are constrained by imprecise intensity modulation, delayed feedback, monotonous training contexts, and limited patient engagement (6). Moreover, the high physical demands on therapists and waning patient motivation preclude the sustained, high-intensity repetitive practice essential for optimal neurorecovery and may inadvertently reinforce abnormal gait (7).

Rehabilitation robots, grounded in motor relearning theory, address these limitations by delivering high-intensity, repetitive, task-specific training with precision and functionality. These devices enable objective quantification of movement parameters, facilitating real-time assessment and individualized treatment optimization (8). As robotic technology advances, rehabilitation robots develop rapidly. They deliver highly repetitive, intensive limb training, promoting functional reorganisation in the brain. This is important for limb function recovery in stroke patients (9, 10). Nevertheless, the efficacy of robot-assisted therapy remains a contentious issue. A 2018 Cochrane review reported positive outcomes (11). More recent trials have largely failed to show significant advantages over conventional rehabilitation (12).

Acupuncture’s role in repairing neurological damage is drawing increasing attention internationally (13, 14). Clinical research indicates that it can alleviate muscle spasticity in stroke patients with hemiplegia, improving limb mobility and coordination, and thereby enhancing daily activity performance (15, 16). Challenges remain: its mechanisms are unclear, and treatment protocols are not standardised, causing inconsistent outcomes. Therefore, its broader application and recognition in modern evidence-based medicine remain limited (17).

For patients with post-stroke motor dysfunction, unimodal rehabilitation is often insufficient to achieve optimal recovery, whereas combined interventions may offer synergistic benefits. Rehabilitation robots deliver repetitive, task-specific training that facilitates motor learning through high-intensity practice and sensory feedback—mechanisms known to indirectly support neuroplasticity (18). However, their effects are primarily mediated peripherally, through motor execution, rather than through direct central neuromodulation. Acupuncture, by contrast, stimulates limb acupoints to generate strong afferent input, broadly activating sensorimotor networks and modulating cortical excitability (19). This complementary “peripheral–central” strategy may synergistically enhance neuroplasticity and optimize functional outcomes after ischemic stroke.

This study aims to clarify the benefits of combining acupuncture with RR therapy in patients with hemiplegia after stroke. To achieve this, a rigorous assessment of the efficacy and safety of the combined therapy for motor function improvement will be conducted through a systematic review and meta-analysis. Ultimately, the goal is to provide strong, evidence-based information to guide clinical decisions.

## 2 Materials and methods

The systematic review protocol was prospectively registered with PROSPERO (CRD420251248863), and the review process and reporting adhered to both the Cochrane Handbook for Systematic Review Authors and the PRISMA (Preferred Reporting Items for Systematic Reviews and Meta-Analyses) statement. The impact of any subgroup analyses performed will be clearly discussed in relation to how they influence the overall findings of the review, consistent with established guidelines for systematic reviews.

### 2.1 Search strategy

A systematic literature search was performed in the following electronic databases: Pubmed, Embase, the Cochrane Central Register of Controlled Trials, Web of Science, Chinese Biomedical Database, Chinese National Knowledge Infrastructure, Wan Fang Data Knowledge Service Platform, and VIP Journal Integration Platform. The databases were searched from inception to November 29, 2025. In addition, we searched the Chinese Clinical Trial Register and ClinicalTrials.gov for ongoing or unpublished trials and screened the reference lists of all included studies and relevant reviews to identify additional eligible studies. The search strategy employed combined controlled vocabulary with free-text terms pertaining to “ischemic stroke,” “hemiplegia,” “acupuncture,” and “rehabilitation robot.” The complete search strategy is detailed in the Supplementary Appendix.^1^

### 2.2 Inclusion criteria

Studies were selected based on the PICOS framework:

1. Population: adult patients (≥18 years) with a clinical and radiological (CT/MRI) diagnosis of ischemic stroke, presenting with hemiplegia or unilateral motor impairment. Only patients in the subacute phase of stroke, defined as 1 week to 6 months after symptom onset.
2. Intervention: acupuncture combined with RR.
3. Comparison: RR or Conventional Rehabilitation alone.
4. Outcomes: Fugl-Meyer Assessment score and Barthel Index.
5. Study design: randomized controlled trials.

### 2.3 Exclusion criteria

Studies were excluded if they met any of the following criteria: (1) duplicate publications; (2) control interventions including any form of true acupuncture; (3) animal studies; (4) reviews; (5) case reports or experience sharing; or (6) unavailable full text.

### 2.4 Study selection and data extraction

Two reviewers independently screened titles, abstracts, and full-text papers according to the eligibility criteria. Disagreements were resolved through discussion or, if needed, by consulting a third reviewer. Both reviewers independently extracted data using a piloted form, including study characteristics, participant demographics, intervention details, outcome measures, and results.

### 2.5 Study quality assessment

Two researchers independently assessed risk of bias using the Risk of Bias 2 (RoB 2) tool following the Cochrane Handbook. Any disagreements were discussed and resolved with the assistance of a third reviewer.

### 2.6 Types of outcomes

The primary outcome was the change in motor function, assessed by the Fugl-Meyer Assessment of Upper Extremity (FMA-UE), the Fugl-Meyer Assessment of Lower Extremity (FMA-LE), and Barthel Index (BI). Secondary outcomes included: activities of daily living (ADL), Stroke-Specific Quality of Life scale (SS-QOL), and the Effective Rate (ER), which refers to the proportion of patients achieving ‘marked improvement’ or ‘cure’ as defined by specific, predefined clinical criteria in the original studies.

### 2.7 Statistical analysis

All statistical analyses were performed using R software (version 4.5.1). For continuous outcomes, the pooled effect was expressed as the mean difference (MD) with a 95% confidence interval (CI). For dichotomous outcomes, it was expressed as the risk ratio with a 95% CI. Heterogeneity was measured using the *I*^2^ statistic. Values of 25, 50, and 75% indicated low, moderate, and high heterogeneity. The significance of pooled effects was assessed using *Z*-tests with a two-sided *p*-value of less than 0.05. Sensitivity analyses were performed using the leave-one-out approach to check robustness. Potential publication bias was assessed for primary outcomes. Visual inspection of funnel plots was complemented by Egger’s linear regression test. A threshold of *p* < 0.10 was used to determine statistical significance for the test. A significant result was interpreted as evidence of potential small-study effects.

## 3 Result

### 3.1 Literature screening

**Figure 1:**
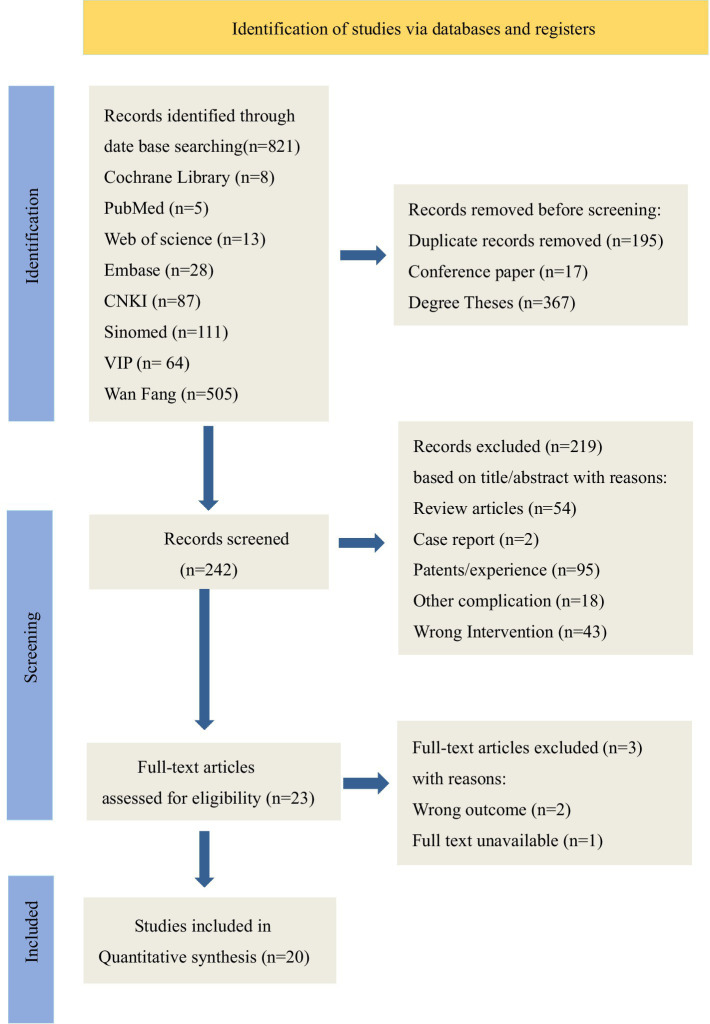
Literature screening flowchart.

A total of 821 documents were retrieved. During title screening, 249 duplicate records, 17 conference proceedings, and 367 academic papers were excluded. In the subsequent PRISMA guideline-based screening of titles and abstracts, studies were excluded if they were (1) systematic reviews; (2) case reports or experiential summaries; (3) focused on other complications; or (4) involved interventions not combining acupuncture with rehabilitation robotics. Ultimately, 20 studies met the inclusion criteria (Figure 1).

### 3.2 Participant characteristics

The analysis included 1,594 participants, equally allocated to the experimental and control groups (each *n* = 797). Across the 11 RCTs, individual study sample sizes ranged from 20 to 60. Mean age did not differ significantly between the groups (*p* > 0.05). Full demographic details are provided in Table 1.

**Table 1:** Participant characteristics.

Trial name	Simple size	Sex (male/female)	Age (years)	Disease course (month)	Intervention group	Control group	Outcomes measures	Adverse reactions
Liu et al. (20)	*I*: 22/22	*I*: 15/7	*I*: 64.28 ± 8.97	*I*: 0.71 ± 0.15	MA + LLRR	CR	SFMA-LE, SS-QOL, ER	Not reported
	*C*: 23/23	*C*: 16/7	*C*: 62.98 ± 8.90	*C*: 0.73 ± 0.16				
Zhang and Ban (30)	*I*: 59/59	*I*: 32/27	*I*: 60.50 ± 5.50	Not reported	MA + LLRR	CR	FMA-LE, FMA-B, BBS, FAC	Not reported
	*C*: 58/58	*C*: 33/25	*C*: 60.5 ± 4.50					
Li (21)	*I*: 33/33	*I*: 17/16	*I*: 59.45 ± 6.18	*I*: 0.82 ± 0.16	MA + ULRR	CR	FMA-UE, MBI, WHO-QOL, ER	Not reported
	*C*: 33/33	*C*: 18/15	*C*: 58.47 ± 6.68	*C*: 0.85 ± 0.15				
Yang et al. (22)	*I*: 34/34	*I*: 21/13	*I*: 45.80 ± 6.90	*I*: 1.47 ± 0.50	MA + ULRR	CR	FMA-UE, FIM, BI	Not reported
	*C*: 34/34	*C*: 17/17	*C*: 46.70 ± 6.60	*C*: 1.50 ± 0.60				
Zhang et al. (31)	*I*: 48/48	*I*: 33/15	*I*: 67.90 ± 3.50	*I*: 3.97 ± 0.95	MA + LLRR	CR	FMA-LE, ADL, BBS, FAC	Not reported
	*C*: 48/48	*C*: 35/13	*C*: 68.40 ± 3.90	*C*: 4.17 ± 0.77				
Li and Wan (32)	*I*: 25/25	*I*: 15/10	*I*: 58.38 ± 15.76	*I*: 1.95 ± 0.96	MA + ULRR	CR	FMA-UE, MBI	Not reported
	*C*: 25/25	*C*: 14/11	*C*: 57.59 ± 15.63	*C*: 1.94 ± 1.01				
Zhu et al. (28)	*I*: 40/40	*I*: 24/16	*I*: 61.27 ± 7.30	*I*: 0.66 ± 0.15	MA + LLRR	CR	FMA-LE, BI, BBS, FAC, TUG	Not reported
	*C*: 40/40	*C*: 23/17	*C*: 60.43 ± 7.44	*C*: 0.66 ± 0.10				
Guo et al. (23)	*I*: 29/29	*I*: 19/10	*I*: 62.20	*I*: 1.19 ± 0.17	MA + LLRR	CR	FMA-LE, FAC, RMS	Not reported
	*C*: 29/30	*C*: 17/12	*C*: 59.40	*C*: 1.08 ± 0.16				
Liu et al. (24)	*I*: 20/20	*I*: 13/7	*I*: 50.55 ± 14.03	*I*: 1.53 ± 0.15	MA + ULRR	CR	FMA-UE, MBI	Not reported
	*C*: 20/20	*C*: 12/8	*C*: 50.20 ± 13.91	*C*: 1.49 ± 0.14				
Jianfei et al. (33)	*I*: 25/25	*I*: 13/12	*I*: 57.90 ± 7.10	*I*: 5.00 ± 2.00	MA + ULRR	ULRR	FMA-UE, MBI, fNIRS	Not reported
	*C*: 25/25	*C*: 15/10	*C*: 58.50 ± 6.30	*C*: 5.60 ± 2.10				
Ma et al. (37)	*I*: 35/35	*I*: 17/18	*I*: 63.11 ± 10.63	*I*: 1.68 ± 0.53	EA + LLRR	LLRR	FMA-LE, BBS, NIHSS, SS-QOL	Not reported
	*C*: 35/35	*C*: 19/16	*C*: 62.36 ± 10.58	*C*: 1.64 ± 0.52				
Chen et al. (25)	*I*: 30/30	*I*: 20/10	*I*: 61.07 ± 5.77	*I*: 0.70 ± 0.12	MA + LLRR	LLRR	FMA-LE, BBS, FAC, MBI	Not reported
	*C*: 30/30	*C*: 18/12	*C*: 59.90 ± 6.13	*C*: 0.68 ± 0.12				
Zhu et al. (28)	*I*: 40/40	*I*: 24/16	*I*: 61.27 ± 7.30	*I*: 0.66 ± 0.15	MA + LLRR	LLRR	FMA-LE, BI, BBS, FAC, TUG	Not reported
	*C*: 40/40	*C*: 21/19	*C*: 60.00 ± 7.48	*C*: 0.62 ± 0.15				
Yang et al. (38)	*I*: 20/20	*I*: 14/6	Not reported	*I*: 0.97 ± 0.13	MA + LLRR	LLRR	FMA-LE, ADL, SCL-90	Not reported
	*C*: 20/20	*C*: 13/7		*C*: 0.97 ± 0.13				
Guo et al. (23)	*I*: 29/29	*I*: 19/10	*I*: 62.20	*I*: 1.19 ± 0.17	MA + LLRR	LLRR	FMA-LE, FAC, RMS	Not reported
	*C*: 28/30	*C*: 17/11	*C*: 58.80	*C*: 1.11 ± 0.16				
Liu et al. (26)	*I*: 53/53	*I*: 31/22	*I*: 69.13 ± 5.84	*I*: 1.38 ± 0.12	MA + ULRR	ULRR	FMA, ARAT, MAS	Not reported
	*C*: 51/51	*C*: 34/17	*C*: 68.38 ± 5.67	*C*: 1.41 ± 0.12				
Zhao et al. (34)	*I*: 30/30	*I*: 18/12	*I*: 52.49 ± 5.06	*I*: 1.47 ± 0.05	MA + ULRR	ULRR	FMA-UE, UEFI, MEP, ADL	Not reported
	*C*: 30/30	*C*: 17/13	*C*: 52.52 ± 4.74	*C*: 1.50 ± 0.04				
Zhang et al. (29)	*I*: 55/55	*I*: 32/23	*I*: 62.44 ± 7.01	*I*: 2.05 ± 0.14	MA + ULRR	ULRR	FMA, sEMG, MBI	Not reported
	*C*: 55/55	*C*: 30/25	*C*: 61.23 ± 6.73	*C*: 2.10 ± 0.20				
Kong (27)	*I*: 30/30	*I*: 15/15	*I*: 62.01 ± 8.93	*I*: 3.52 ± 1.07	MA + ULRR	ULRR	FMA-UE, MBI, IEMG, ER	One case of gastrointestinal discomfort
	*C*: 30/30	*C*: 17/13	*C*: 61.36 ± 8.42	*C*: 3.46 ± 0.94				
Wang et al. (39)	*I*: 60/60	*I*: 36/24	*I*: 52.60 ± 6.30	Not reported	EA + ELRR	LLRR	FMA-LE, BBS, FAC, SSR	Not reported
	*C*: 60/60	*C*: 38/22	*C*: 53.90 ± 6.00					
Shen et al. (35)	*I*: 30/30	*I*: 19/11	*I*: 59.87 ± 3.21	*I*: 1.52 ± 2.52	EA + ELRR	ULRR	FMA-UE, ER, NIHSS, STEF, BBS	Not reported
	*C*: 30/30	*C*: 18/12	*C*: 59.89 ± 3.18	*C*: 1.56 ± 2.50				
Lan et al. (36)	*I*: 50/50	*I*: 27/23	*I*: 57.30 ± 8.15	*I*: 1.17 ± 0.36	MA + LLRR	LLRR	FMA-LE, MAS, MBI, ER	Not reported
	*C*: 50/50	*C*: 26/24	*C*: 56.16 ± 7.61	*C*: 1.16 ± 0.36				

MA, manual acupuncture; EA, electroacupuncture; LLRR, lower-limb rehabilitation robot; ULRR, upper-limb rehabilitation robot; CR, conventional rehabilitation; SFMA-LE, simplified Fugl-Meyer Assessment of Lower Extremity; FMA-LE, Fugl-Meyer Assessment of Lower Extremity; FMA-UE, Fugl-Meyer Assessment of Upper Extremity; SS-QOL, Stroke-Specific Quality of Life scale; BBS, Berg Balance Scale; ADL, activities of daily living; BI, Barthel Index; MBI, Modified Barthel Index; ER, Effective Rate; FIM, Functional Independence Measure; FAC, Functional Ambulation Category; RMS, Root Mean Square; fNIRS, Functional Near-Infrared Spectroscopy; TUG, Timed Up and Go test; SCL-90, Symptom Checklist-90; ARAT, Action Research Arm Test; MAS, Modified Ashworth Scale; sEMG, Surface Electromyography; SSR, Sympathetic Skin Response; STEF, Simple Test for Evaluating Hand Function.

### 3.3 Risk of bias in the studies

**Figure 2:**
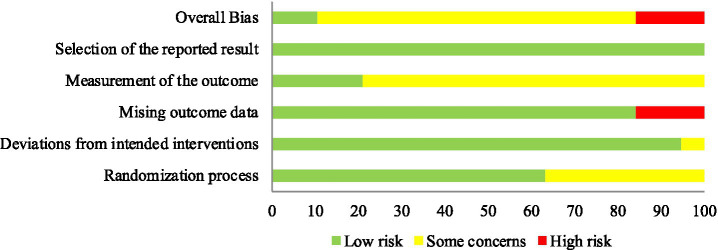
Risk of bias summary.

**Figure 3:**
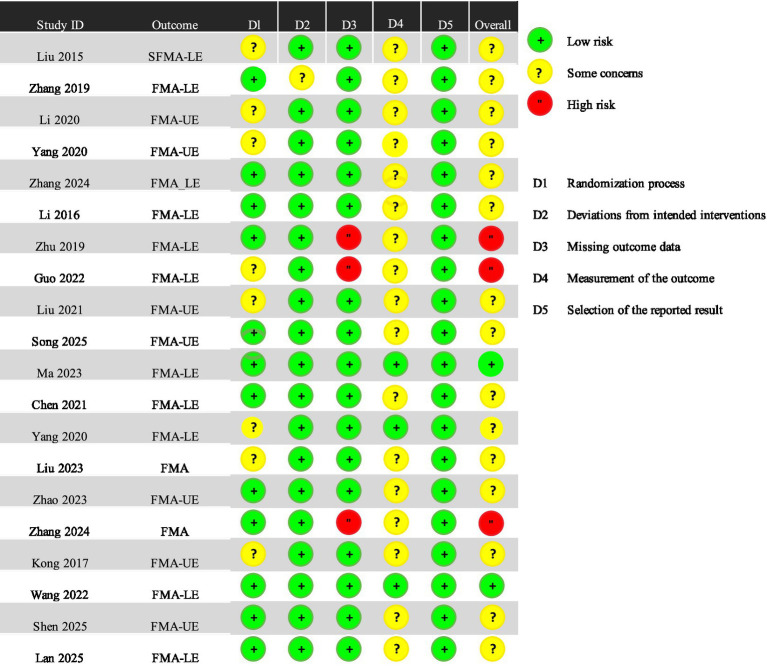
Risk of bias graph.

The summary of risk of bias in the included RCTs, assessed using the Cochrane RoB 2 tool, is presented in Figure 2. Most studies did not indicate a significant risk of bias in the randomisation process. However, eight trials (20–27) mentioned only the term ‘random’ without specifying the method for generating random sequences. These studies were therefore assessed as having an unclear risk of bias. Among the 20 studies, three (23, 28, 29) were assessed as having a high risk of missing outcome data because they did not perform analyses for cases lost to follow-up. Seventeen studies (20–28, 30–36) raised concerns regarding the measurement of outcomes. In these studies, assessors were aware of the intervention received by participants, which could potentially influence outcome evaluation. All eligible studies were deemed to have a low risk of bias in the selection of reported outcomes (Figure 3).

### 3.4 Synthesis results

#### 3.4.1 FMA-UE

**Figure 4:**
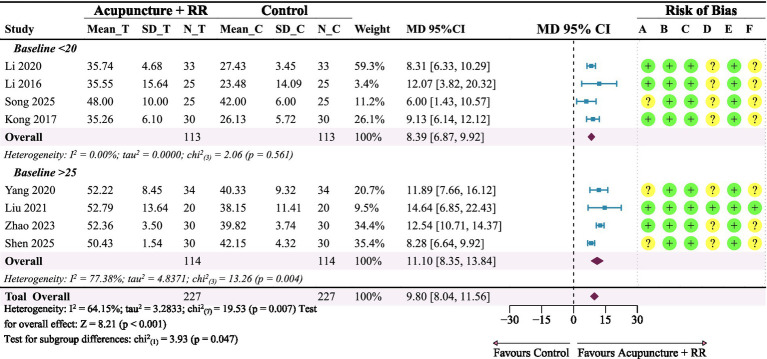
Forest plot for FMA-UE. Combined therapy demonstrates a trend toward improved FMA-UE scores in IS patients.

**Figure 5:**
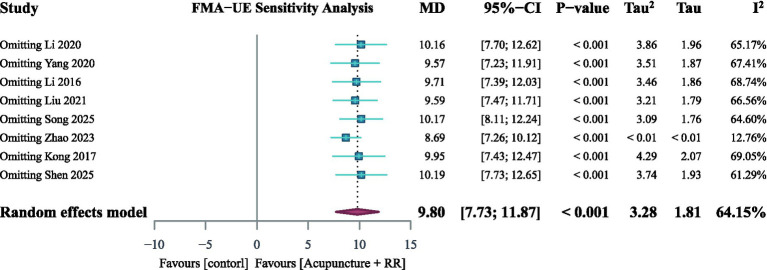
Sensitivity analysis found no notable abnormalities when studies were sequentially excluded.

A total of eight studies (21, 22, 24, 27, 32–35) reported FMA-UE. Meta-analysis results showed that acupuncture combined with RR therapy significantly improved FMA-UE scores in IS patients compared with the control group, with a statistically significant difference (MD = 9.80, 95% CI: 8.04–11.56; *p* < 0.001), with moderate heterogeneity among studies (*I*^2^ = 64.15%) (Figure 4). Sensitivity analyses indicated that excluding any single study did not alter the direction or significance of the pooled effect size, suggesting robust conclusions. Heterogeneity analysis suggested that Zhao et al. (34) may have been the primary source of heterogeneity; however, excluding this study did not change the conclusions (Figure 5). To further explore sources of heterogeneity, we conducted subgroup analyses based on patients’ baseline FMA-UE scores. In the subgroup with poorer baseline function, combined therapy demonstrated a clear and consistent improvement (MD = 8.39, 95% CI: 6.87–9.92; *I*^2^ = 0%). In contrast, for the subgroup of patients with better baseline function, although the point estimate indicated a larger effect (MD = 11.10, 95% CI: 8.34–13.84), the stability of this result was low due to extremely high heterogeneity between studies (*I*^2^ = 77.38%) (Figure 4).

#### 3.4.2 FMA-LE

**Figure 6:**
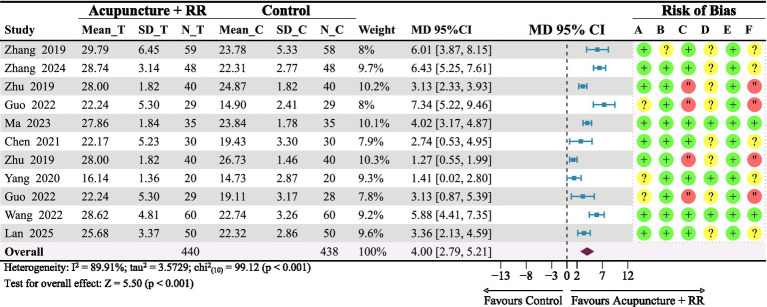
Forest plot for FMA-LE. Combined therapy is associated with increased FMA-LE scores among patients with IS.

**Figure 7:**
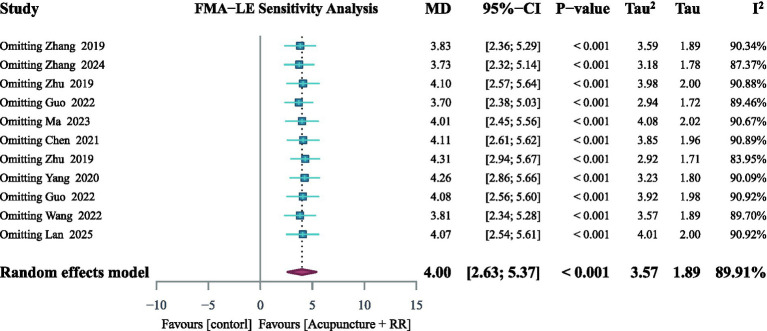
Sequentially excluding studies in the sensitivity analysis revealed no significant abnormalities.

**Figure 8:**
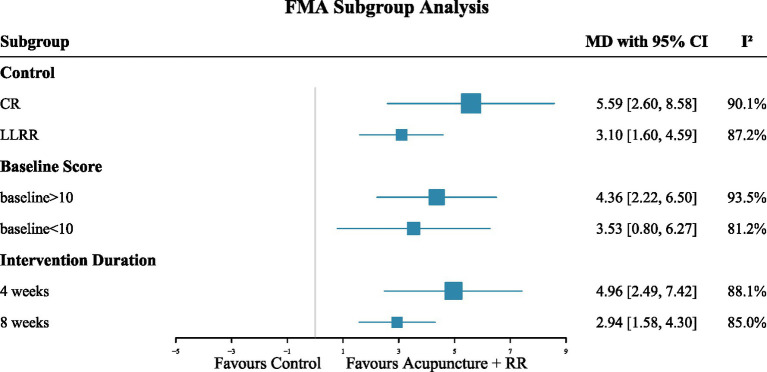
Subgroup analysis of the effect of combination therapy on FMA-LE.

Nine studies (23, 25, 28–30, 36–39) reported on the FMA-LE. A random-effects meta-analysis revealed that combined therapy significantly improved FMA-LE scores in IS patients compared with the control group, with a statistically significant difference (MD = 4.00, meaning mean difference; 95% CI: 2.79–5.21; *p* < 0.001) (Figure 6). However, substantial heterogeneity existed between studies. To assess the robustness of the pooled results, we conducted a sensitivity analysis by sequentially excluding individual studies. Results indicated that removing any single study did not alter the direction or statistical significance of the effect size, suggesting that the primary conclusions were robust (Figure 7). To explore potential sources of heterogeneity, subgroup analyses were conducted based on baseline FMA-LE scores, stroke phase, and intervention duration. Findings demonstrated that combined therapy consistently produced significant improvements in FMA-LE (Figure 8).

#### 3.4.3 Daily functions

**Figure 9:**
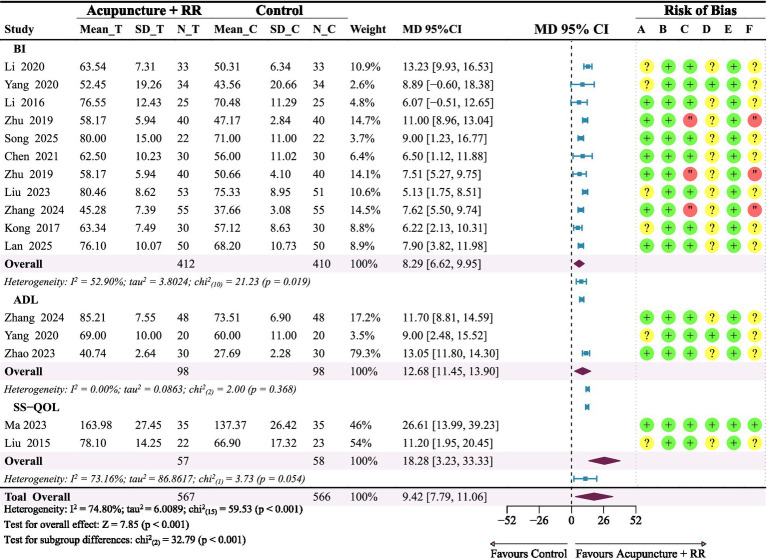
Forest plot of daily functions. Combined therapy appears to benefit daily function scores in patients with IS.

A total of 15 studies (20–22, 25–29, 31–34, 36–38) utilised three distinct scales to assess the daily functional capabilities among stroke patients. To provide a comprehensive evaluation of intervention efficacy, we conducted subgroup analyses by scale type and calculated overall pooled effects. Specifically, the BI subgroup comprised 11 studies, in which meta-analysis revealed that combined therapy significantly improved BI scores in stroke patients (MD = 8.29, 95% CI: 6.62–9.95). In contrast, the ADL subgroup included three studies, and its meta-analysis indicated that combined therapy significantly improved ADL scores in IS patients (MD = 12.68, 95% CI: 11.45–13.90). Furthermore, the SS-QOL subgroup consisted of two studies and demonstrated significant improvement in SS-QOL scores in IS patients who received combined therapy (MD = 18.28, 95% CI: 3.23–33.33). Bringing together all 16 studies, the overall pooled analysis revealed that combined therapy significantly improved the ability to perform activities of daily living in IS patients (MD = 9.42, 95% CI: 7.79–11.06; *p* < 0.001). Notably, differences between subgroups were statistically significant (*p* < 0.0001), suggesting that the type of scale used was a key source of heterogeneity (Figure 9).

#### 3.4.4 ER

**Figure 10:**
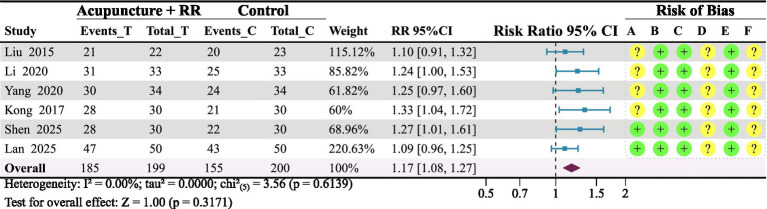
Forest plot of ER status. Combined therapy shows a favourable trend in improving ER status among IS patients.

Six studies (20–22, 27, 35, 36) reported on ER. To further assess the combined therapy’s impact, the meta-analysis demonstrated that combination therapy significantly improved ER in IS patients (relative risk = 1.17, 95% confidence interval: 1.08–1.27; *p* < 0.001) (Figure 10).

### 3.5 Publication bias

**Figure 11:**
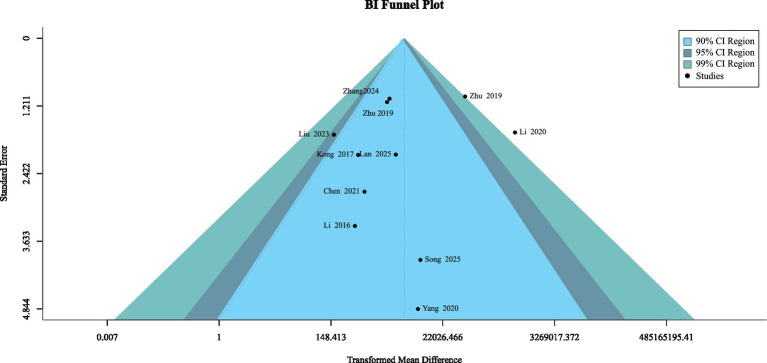
Visual inspection of publication bias using a funnel plot.

Using a fixed-effects model to analyse funnel plots, an inverted funnel plot was generated when the sample size exceeded ten. The funnel plot depicted in Figure 11 exhibits a symmetrical pattern. All *p*-values from the Egger regression test and Begg’s funnel plot asymmetry test were >0.05, indicating no significant publication bias.

## 4 Discussion

### 4.1 Principal findings

This study is the first review to use meta-analysis to assess the effectiveness of acupuncture and rehabilitation robots in treating hemiplegia following a stroke. Results show that combining these treatments clearly improves patients’ movement and daily activities more than traditional manual therapy or robotics alone. The combination also increased treatment Effective Rate. Further breakdowns showed that robotic therapy helped motor recovery, regardless of the patients’ starting point or whether the therapy lasted 4 or 8 weeks. Notably, the improvement in lower extremity function (FMA-LE: MD = 4.00) was relatively modest, prompting us to assess its clinical relevance against established thresholds. Published estimates of the minimal clinically important difference for FMA-LE in stroke survivors range from 4 to 6 points, depending on stroke phase and definition of meaningful change (40). Our pooled estimate meets the lower bound of this range, suggesting that even these more modest gains in lower limb function are likely clinically perceptible.

### 4.2 Comparison to prior work

Turning to prior research, as the first systematic review and meta-analysis evaluating the efficacy of acupuncture combined with RR therapy for hemiplegia following IS, this study fills an evidence gap in the field. Compared with previous single interventions, the combined therapy demonstrated significant advantages in improving patients’ motor function. Existing evidence indicates that while monotherapies demonstrate efficacy, their impact on motor function improvement remains relatively limited: robot-assisted gait training alone yielded a 2.10-point improvement in lower limb motor function (FMA-LE: MD = 2.10) (41); upper limb rehabilitation robotics produced outcomes comparable to conventional rehabilitation (FMA-UE: MD = 0.696) (42); the effect size of acupuncture alone on lower limb motor function is also modest (FMA-LE: MD = 0.48) (43).

Beyond motor recovery, the fundamental goal of stroke rehabilitation is to restore functional independence and quality of life. Previous meta-analyses have shown that monotherapies confer significant but limited benefits in this domain: robot-assisted therapy alone improved activities of daily living, with effect sizes ranging from moderate for overall ADL (MD = 7.80, 95% CI: 4.15–11.45) (44) to more modest for Barthel Index (MD = 4.70, 95% CI: 0.98–8.41) (45); acupuncture monotherapy yielded comparable improvements [ADL: MD = 5.99, 95% CI: 5.33–6.64 (46); and BI: MD = 4.59, 95% CI: 1.43–7.75 (47)]. In marked contrast, our meta-analysis demonstrated that combined acupuncture and robot-assisted therapy produced substantially greater gains across multiple measures of daily living: Barthel Index (MD = 8.29, 95% CI: 6.62–9.95), activities of daily living scale (MD = 12.68, 95% CI: 11.45–13.90), and Stroke-Specific Quality of Life scale (MD = 18.28, 95% CI: 3.23–33.33)—all indicating clinically meaningful improvements in real-world function. Moreover, combination therapy significantly increased the proportion of patients achieving marked improvement or cure (ER: RR = 1.17, 95% CI: 1.08–1.27), suggesting that observed motor gains translated into clinically recognizable recovery. Collectively, these findings reinforce the clinical value of the combined approach, demonstrating that enhanced motor function facilitates greater independence and perceived recovery—outcomes of paramount importance to patients and caregivers.

Acupuncture combined with RR may prove effective in post-stroke hemiplegia. Its mechanism may stem from a synergistic promotion of neural plasticity at the ‘peripheral drive-central regulation’ level. RR acts as a standardised peripheral sensory-motor driver. It delivers high-intensity, repetitive task training with real-time feedback (48, 49). This process inputs substantial, regularised motor learning signals to the central nervous system. As a result, it helps reorganise the motor cortex and restore interhemispheric balance (50). Acupuncture therapy primarily regulates the functional state of the central nervous system. It transmits peripheral sensory input to modulate the excitability of the spinal cord and cortex (51). It also regulates the release of neurotrophic factors and balances neurotransmitter systems, such as GABA and glutamate (52, 53). This improves the neural microenvironment at the molecular and network levels.

### 4.3 Strengths and limitations

This meta-analysis possesses several strengths. It adheres to rigorous Cochrane and PRISMA guidelines. This ensures transparency and reproducibility. A comprehensive, unrestricted search strategy across multiple databases minimises the risk of missing relevant studies. By including only randomised controlled trials, this review provides high-quality evidence on the efficacy of combined interventions. Independent, duplicate data extraction and quality assessment enhance reliability. Subgroup analyses based on baseline FMA scores, intervention duration, and control methods provide insight into effect modifiers. Publication bias assessment for primary outcomes further strengthens these conclusions.

This study acknowledges certain limitations. Firstly, as the risk of bias assessment tool RoB2 was employed for rigorous quality evaluation, only two studies were deemed to be at low risk, thereby limiting the robustness of our conclusions. Secondly, despite conducting multiple subgroup analyses, we were unable to clearly identify the primary cause of high heterogeneity in the FMA-LE outcomes. Thirdly, the study did not analyse patient follow-up data. This precluded assessment of the long-term efficacy of this combined therapy, which may impact the study’s conclusions. Finally, although all patients met the predefined subacute criterion (1 week to 6 months post-stroke), the subacute phase itself encompasses substantial heterogeneity across the early-to-late recovery continuum. Due to limitations in the available data, we could not stratify outcomes by precise timing within this window.

### 4.4 Future directions

Future research should address key limitations through several complementary approaches. First, large-scale, methodologically rigorous randomized controlled trials are required to strengthen the evidence base. Such studies must report standardized combined intervention protocols, with strict adherence to CONSORT and STRICTA guidelines. Complete implementation and transparent reporting of allocation concealment and blinding are essential, and trials should stratify patients by early versus late subacute phases to determine whether treatment timing moderate efficacy. Second, beyond validating immediate therapeutic effects, long-term follow-up is needed to assess durability of recovery. Third, incorporating neuroimaging or electrophysiological techniques could elucidate the neural mechanisms underlying synergistic actions of combined therapy. Collectively, these efforts will advance the field of stroke rehabilitation.

## 5 Conclusion

This study highlights the promising potential of acupuncture combined with robotic therapy for improving limb motor function, activities of daily living, and efficacy rates in patients with hemiplegia following stroke. However, future research needs large-scale, methodologically rigorous randomised controlled trials. This will enhance the quality of evidence.

## Data Availability

The original contributions presented in the study are included in the article/supplementary material, further inquiries can be directed to the corresponding authors.

## Footnotes

1
https://www.crd.york.ac.uk/PROSPEROFILES/986da3335617240664523860dfbf84ce.pdf
